# Educating medical trainees on medication reconciliation: a systematic review

**DOI:** 10.1186/s12909-015-0306-5

**Published:** 2015-03-07

**Authors:** Aliya Ramjaun, Monisha Sudarshan, Laura Patakfalvi, Robyn Tamblyn, Ari N Meguerditchian

**Affiliations:** 1 McGill Clinical and Health Informatics Research Group, McGill University, 140 Pine Avenue West, Montreal, Canada; 2 Department of Surgery, McGill University Health Centre, Montreal, Canada; 3 Department of Epidemiology and Biostatistics, McGill University, Montreal, Canada; 4 Department of Oncology, McGill University Health Centre, Montreal, Canada

**Keywords:** Medication reconciliation, Patient safety, Patient discharge, Medical education

## Abstract

**Background:**

Effective medication reconciliation is critical in reducing the risk of preventable adverse drug events. Medical trainees are often responsible for medication reconciliation on admission, transfer and discharge of the most vulnerable patients; therefore, it is important that trainees are educated on this aspect of quality care.

**Methods:**

We conducted a systematic review using MEDLINE and EMBASE databases to identify education initiatives targeted at improving trainee skill and knowledge in carrying out medication reconciliation. Studies published in English or French between July 1980 and July 2013, where the primary focus of the article was the role of medical trainees in conducting medication reconciliation, and where trainee-specific data was reported, were included. Included articles must have reported trainee-specific data. Given the anticipated heterogeneity and array of outcomes, we were unable to employ a specific tool in assessing the risk of bias across studies.

**Results:**

Seven studies met pre-specified eligibility criteria, indicating the lack of published education initiatives targeted towards improving trainee knowledge and experience. Four described an education intervention targeted towards students completing internal medicine clerkship, while the remaining 3 were implemented among residents. Although no two interventions were the same, 5 out of 7 included an experiential component.

**Conclusions:**

Varying success was achieved with medication reconciliation education interventions. While some noted improved competence and/or confidence amongst trainees, namely undergraduate medical students, others noted little effect resulting from the intervention.

## Background

Up to a quarter of admissions to acute care hospitals are related to adverse drug events (ADE) [1,2]. In addition to prolonged hospitalizations and unnecessary complications [3], ADEs result in a significant number of patient deaths, estimated at over 7000 per year in the US [4]. ADEs are also associated with an avoidable financial burden for healthcare institutions, estimated at $3.5 million US dollars per year per hospital [5]. Across the United States, between 1995 and 2000, this has translated into an increase in costs associated with ADEs from an estimated $76.6 billion to $177.4 billion [2].

A large proportion of ADEs are preventable. According to a 2007 report by the Institute of Medicine, over 400,000 preventable ADEs occurred in the United States over the course of a year [5]. This is typically a result of incomplete drug information, prescribing and dispensing errors, as well as the overuse or underuse of medications [6,7]. These types of inconsistencies in medication history are seen in at least 67% of hospital admissions [8-13]. In the observational study conducted by Witherington et al., in which the frequency of incomplete discharge information was assessed in emergency department users, approximately two-thirds of all discharge documents were incomplete with regards to changes in medication [14]. Recently discharged patients are also at risk, as an estimated 72% of ADEs after discharge are related to a lack of proper medication reconciliation [6,15]. In fact, 70% of hospital readmissions within 30 days are related to an ADE [16,17].

Effective medication reconciliation is critical in reducing the risk of preventable ADEs. According to the Institute for Safe Medication Practices (ISMP), medication reconciliation requires that healthcare professionals collaborate with patients, families and care providers to ensure accurate and comprehensive medication information is consistently communicated across transitions of care. This involves performing a systematic and comprehensive review of all the medications a patient is taking, to ensure that medications being added, changed or discontinued are carefully evaluated [18]. Successful execution of medication reconciliation may reduce potentially avertable ADEs and decrease mortality and morbidity [19,20]. Hospital accreditation bodies in North America, have therefore made medication reconciliation processes a mandatory requirement [21,22].

Despite this, compliance with optimal medication reconciliation protocols is poor, with the process being performed for less than 20% of patients in many institutions [3,23]. Medication reconciliation entails multidisciplinary involvement of nurses and pharmacists, however it is ultimately the physician’s responsibility to validate the complete medication history, and formulate and finalize admission and discharge prescriptions. In the case of teaching hospitals, this duty is most often carried out by physicians-in-training (fellows, residents or medical students). Few medical education programs, however, have incorporated safe patient transitioning and medication reconciliation into their curricula [24]. The objective of this systematic review is to identify studies, of any type, reporting on educational interventions aimed at improving the knowledge and skill of medical trainees (i.e. medical students, residents) in carrying out effective medication reconciliation, and to determine which educational interventions are most effective.

## Methods

### Systematic search strategy

A systematic search of the medical literature was conducted in July 2013. This involved searching electronic databases, including MEDLINE and EMBASE, for articles published between 1980 and 2013. For MEDLINE (Appendix), the following Medical Subject Headings (MeSH) were used: (“Medical Records Systems, Computerized” [MeSH] or “Medication Errors” [MeSH] or “Medication Reconciliation” [MeSH] or “Continuity of Patient Care” [MeSH] or “Medication Systems, Hospital” [MeSH]) AND (“Drug Prescriptions” [MeSH] or “Patient Discharge” [MeSH]) AND (“Students, Medical” [MeSH] or “Education, Medical, Graduate” [MeSH] or “Education, Medical, Continuing” [MeSH], or “Education, Medical, Undergraduate” [MeSH] “Internship and Residency” [MeSH]). An analogous search was reproduced for use of the EMBASE database. The reference lists of relevant articles were also searched using Scopus to capture all possible studies.

### Study selection

After search completion, duplicate entries were removed. The remaining records were independently screened by two reviewers (A.R. and L.P.) and irrelevant articles were excluded. Studies published in a language other than English or French, editorials and reviews were excluded. In accordance with study protocol, available on request, we included all other publication types of any quantitative or qualitative design, including conference proceedings captured through electronic searching, so as to improve coverage of the grey literature. Studies were included if the primary focus of the article was on an educational intervention used to improve medication reconciliation competency and/or skills, specifically targeting medical trainees (medical students and residents). When reporting on an intervention carried out in an academic setting, studies reporting exclusively on the impact of an intervention without trainee-specific data were excluded. Likewise, studies discussing the role of medical trainees in reducing post-discharge adverse events, without any specific mention of medication reconciliation were discarded.

A piloted form was created and used to extract data by two reviewers (A. R. and L.P.). A third reviewer (A.M) resolved any disagreements. For each eligible study, data was collected on study characteristics, including the number of participants per intervention, type of intervention, level of training of students and specialty, as well as the outcomes measured.

## Results

**Figure 1:**
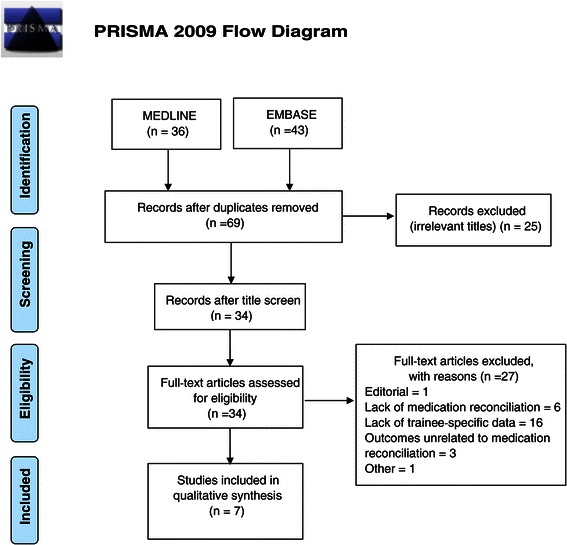
**PRISMA flow diagram of study selection.**

A total of 69 studies were identified from the search after removing duplicates. These were screened by title and/or abstract and 25 irrelevant records were excluded. Subsequently, 34 full text articles were reviewed, from which 7 were found to be eligible for inclusion in the qualitative synthesis (Figure 1). A quantitative analysis by way of a meta-analysis was deemed inappropriate in light of the considerable heterogeneity across studies with regards to study populations and study designs. Study characteristics are summarized in Table 1. All of these studies were conducted in the United States. Of these 7 studies, 6 described an education initiative designed to improve medical trainees’ understanding of and/or ability to confidently carry out medication reconciliation. The remaining study primarily assessed how the involvement of trainees in the post-discharge patient care trajectory could improve quality of care, as well as trainee competence in performing medication reconciliation [25].

**Table 1:** **Study characteristics**

Author; year	N	Intervention	Experimental or curriculum-integrated	Level of training	Specialty	Outcomes measured	Results
Bray-Hall et al. 2010 [26]	98	Interactive group sessions, self-directed learning exercises, patient interaction at discharge and home visit	Curriculum-integrated	Undergraduate clerkship	Internal medicine	Confidence in transitional care (5-point scale)	Increased confidence, 2.7 to 4.0 (p<0.01)
Mann et al. 2011 [30]	226	15 minute standardized patient discharge encounter	Experimental	Residency	Various	Proportion of high performers in discharge counselling domains (includes medication reconciliation)	37.2% of participants considered high performers in medication reconciliation. Residents from programs with primary care component had more high performers (p=0.031)
Morgan-Gouveia et al. 2013 [28]	78	Reflective exercise and transitions in care workshop	Experimental	Undergraduate clerkship	Internal medicine	N/A (qualitative study)	N/A
Ouchida et al. 2009 [29]	103	Interdisciplinary lectures, educational video, small-group discussion and team-based learning exercise	Curriculum-integrated	Undergraduate clerkship	Internal medicine	Knowledge, attitude, behaviours within domains of transitional care	Significant improvements for all domains assessed
Weisman et al. 2012 [25]	20	Resident phone call within 72 hours of discharge	Experimental	Residency	Internal medicine	Attitude and competence	No change
Young et al. 2011 [31]	13	Home visit to recently discharged patient	Experimental	Residency	Internal medicine	Confidence in transitional care	No change for medication reconciliation skills
Lindquist et al. 2008 [27]	158	Lecture from pharmacist, medication reconciliation simulation	Curriculum-integrated	Undergraduate Year 2	N/A	Knowledge/ comfort obtaining medication histories	Increase of 27% (p<0.01) in knowledge and increase of 20% (*p*<.001) in comfort level

In the 6 studies that described an education or training program aimed at improving medical trainees’ knowledge and skill in performing effective medication reconciliation, a variety of teaching methods were utilized. While some interventions were targeted towards educating medical students, others were more geared towards medical residents.

### Educating medical students on medication reconciliation

Four of the 7 seven studies obtained through our systematic search described education initiatives aimed towards improving medication reconciliation practices amongst medical students (Table 1). In the study conducted by Bray-Hall et al., third year medical students first attended introductory didactic sessions, which provided students with foundational knowledge pertaining to medication reconciliation and safe patient transitioning from healthcare settings [26]. Two faculty members then demonstrated through role-play an ideal peri-discharge meeting between a patient and student. This involved educating the patient on their disease, medications, symptoms requiring attention, and follow-up care [26]. Students then engaged in self-study, experiential learning involving direct patient care, and small-group exercises in which they discussed clerkship experiences and reflected on the experiential aspects of the education program. A similar approach was used by Lindquist et al., where the authors implemented an interactive learning exercise to educate second year medical students on medication reconciliation [27]. This involved first receiving instruction from a pharmacist on how to accurately obtain a patient’s medication history, as well information on relevant tools and sources that may be used throughout the process. With the aid of a standardized patient (SP), the teaching pharmacist subsequently demonstrated how to initiate a medication history according to a script of key questions. With a partial medication list now available, the students continued the medication history-taking and/or reconciliation process in small groups (3–4 people per group), after which the entire class reconvened to discuss and reflect on their findings [27].

Interestingly, the results of both studies indicated that following these training exercises, students experienced an increase in confidence in establishing a patient’s medication history and/or conducting medication reconciliation [26,27]. In the study conducted by Bray-Hall et al., specifically, based on the results of a questionnaire assessing student confidence prior to and following the clerkship exercise, overall confidence scores increased from an average of 2.7 out of 5 (SD = 1.0) to 4.0 out of 5 (SD = 0.8) (p < 0.01) [26]. As part of the experiential learning exercise in which students carried out post-discharge visits at the patient’s home, hospice or skilled-nursing facility, students were able to identify medication discrepancies in 43% of the visits. In evaluating the program administered by Lindquist et al., students also completed a survey in which they rated their knowledge level as having increased by 27% (p < 0.001), and their comfort in performing medication reconciliation as having increased by 20% (p < 0.001) [27].

Although all of the education initiatives described thus far have incorporated an experiential learning component, the findings of the study conducted by Morgan-Gouveia et al. (2013) indicate that this may not always be necessary. The education intervention described in this study required that third-year medical students attend a 3-hour workshop as a part of their internal medicine clerkship [28]. As a part of the workshop, they participated in 5 interactive small-group sessions in which the key skills needed in ensuring safe healthcare transitions were taught using a case-based approach. Based on the results of a pre- and post-workshop survey focused on assessing students’ independence in carrying out 10 discharge tasks, including medication reconciliation, students reported significant increases in independence in conducting medication reconciliation. Where students previously reported, according to a 4-point scale, a mean score of 2.7 out of 4, after the workshop the mean score was 3.2 (p < 0.01) [28]. Student participants also felt they could more independently educate patients at discharge, review discharge instructions and communicate with the outpatient provider, among other discharge activities [28]. Ouchida et al. also carried out a similar case-based education intervention coupled with small-group exercises involving multidisciplinary instructors. There was not, however, a significant growth in the number of students performing medication reconciliation [29]. It is also important to note that none of these studies assessed more objective outcomes such as increase in skill or accuracy in performing medication reconciliation.

### Educating medical residents on medication reconciliation

Experiential teaching methods have also been used in educating medical residents in medication reconciliation (Table 1). As a part of the study conducted by Mann et al., 226 residents engaged in a 15-minute encounter with a standardized patient (SP) for which they were instructed to counsel a soon-to-be discharged patient [30]. SPs evaluated the quality of the residents’ performance. In addition to certain professional domains (i.e. verbal professional demeanor), the residents were also assessed for whether or not they provided disease education, facilitated patient understanding, follow-up care plans and medication reconciliation. The results of the SPs’ evaluations were then used to identify high performers with respect to each of the domains. Although most residents (79%) felt that the encounter was realistic and 61% reported increased confidence as a result of the exercise, only 37.2% of the residents were considered ‘high performers’ in carrying out effective medication reconciliation. Interestingly, residents from programs with a primary care component were significantly more likely to be high performers in this area compared to other residents.

Like Mann et al., other groups have also opted to educate residents on medication reconciliation using experiential teaching methods. This includes the study conducted by Young et al., in which residents participated in a “Hospital to Home” program. This involved visiting a home or nursing home to follow-up with a patient that they had recently cared for in hospital [31]. In assessing how well residents had grasped certain key skills necessary in safely discharging a patient, including medication reconciliation, all participating residents felt they could perform these tasks either ‘fairly well’ or ‘quite well’. When comparing their ability to perform certain discharge tasks before and after the intervention however, participating residents stated that their skills in conducting medication reconciliation had not significantly improved. Similar results were obtained by Weisman et al., where they found that having a resident make a phone call to recently discharged patient had no appreciable impact on residents’ competence in performing medication reconciliation and related tasks [25]. This study did not provide additional information on supervision or formative feedback from trainees.

## Discussion

The process of medication reconciliation is described as having three important elements: verification (obtaining the most up-to-date medication list), clarification (determining current dosage, utilization and adherence) and reconciliation (deciding on required changes and ensuring that this information is available to other treating physicians [32]. Although effective medication reconciliation represents a critical component of safe transitioning from or between healthcare settings, medical trainees have traditionally received little or no formal training and/or tools for eliciting a proper medication history, review and reconciliation [33]. The results of our systematic review also indicate that a limited number of education initiatives have been targeted towards improving medication reconciliation skills amongst trainees. We found that only 3 out of the 7 studies had reported on an education initiative that had been formally integrated into a given undergraduate or post-graduate medical education curriculum.

Organizations such as the Accreditation Council for Graduate Medical Education, however, have recognized effective medication reconciliation as an important aspect of quality improvement and emphasized that it be an educational goal [34]. In response, groups such as Atlantic Health have sought to enhance trainee competency through “Systems-Based Practice and Practice-Based Improvement” across a variety of residency programs [34]. These sessions consisted of didactic teaching, as well as training on implementing and improving medication reconciliation within residency programs. Each residency program subsequently set goals, tracked their results and made improvements accordingly. Multidisciplinary expert faculty provided guidance and program directors monitored progress. At the end of the collaborative, almost all programs had made significant changes to improve medication safety. Examples of quality improvement programs included a “No pass rule” in surgery, which did not permit the surgeon to proceed in the operating room without a completed medication reconciliation form ensuring accurate postoperative orders. Much of the success of this quality initiative can be attributed to the specialty-specific goals set by each residency program.

### Obstacles in medication reconciliation education

While the practice of medication reconciliation represents a key skill to be learned by medical trainees, a number of obstacles have remained unaddressed. Given recent reforms in resident work hours [35] and the impact this has had on continuity of care as provided by individual residents, integrating training and implantation of medication reconciliation often poses a challenge. Observational studies have indicated that interns and junior residents spend a significant amount of time on administrative duties compared to their senior counterparts, and therefore, cultivating medication reconciliation as an essential part of patient care and not a mundane documentation duty is essential [36,37]. That being said, educational efforts should be devoted to changing the perception trainees have towards effectively completing medical documentation, including medication reconciliation. In addition, the nature of residency training requires rotations in a multitude of training sites. This constant displacement among institutions with different medication reconciliation processes can be challenging when coordinating the effective implementation of strategies for reduction in adverse drug events attributable to medication reconciliation failures.

Certain barriers also stem from resident attitudes or personal factors, as well as environmental and system-related issues [38]. Personal factors include a decreased level of involvement in error recognition and reduction and personal responsibility [38-40]. Due to the nature of training with constant shift of rotations, institutions, and lack of long-term follow-up with patients, these attitudes often stem from residents viewing themselves as transient care providers [38]. The most commonly reported impediment to error identification was environmental, defined as the culture of the organization or institution. This is reflected mainly by concerns about perceived legal repercussions and the impact of error identification on career development [41]. Much in the way that other professions have encouraged error reporting by creating a safe environment for discussion, medical education programs may benefit from the implementation of such a program or forum. This may take place in the form or morbidity or mortality rounds, where trainees can openly discuss, without fear for legal repercussions, their experiences and how certain adverse events may be avoided in future. System-related issues revolve around organizational structure and included resident inexperience coupled with a lack of a defined structured process for error reporting and reduction [24,27,39].

### Involving trainees in developing patient safety initiatives

In recognizing the importance of contextualizing and managing medication-related safety from the systems level [42], a number of reports have called for the involvement of house staff when developing patient safety initiatives. Given that in academic centers discharge is most often completed by physicians-in-training [43], these reports outline the importance of resident involvement in each step of a quality improvement process from participating in committee decisions to carrying out such initiatives at the bedside. However, only recently have studies been conducted to evaluate the feasibility and impact of house staff involvement in planning and executing medication reconciliation measures. Evans et al., for example, studied the implementation of a novel initiative to involve residents in quality improvement measures including medication reconciliation [44]. A House Staff Quality Council (HQC) was formed and residents were elected from different departments to form the council. In addition to meeting monthly, the HCQ worked closely with hospital administrators and policy development to contribute and implement quality care initiatives. The HCQ was also involved in communicating the medication reconciliation initiatives and changes to house staff not directly involved in the council. Compliance with reconciliation rose from 30-52% at some sites to over 90% at 6 months post-intervention. The results suggest that resident input in the implementation of medication reconciliation interventions is essential to ensure the success of the program.

According to another study conducted in a large academic health centre, prior consultation with residents dramatically changed the format of the form used for medication reconciliation [45]. Specifically, suggestions and recommendations from house staff rendered the worksheet more feasible, easy to use and practical for nurses and residents alike. This facilitated rapid adoption and good compliance rates, which lead to a 43% reduction in ADEs [45]. Furthermore, regular feedback was included, to improve the process which allowed all residents to be an active part of improving medication reconciliation. These studies provide salient evidence suggesting that implementing medication reconciliation systems in health care institutions is not a process limited to hospital administrators and staff physicians. Direct care providers such as residents can provide invaluable input and regular feedback that can improve practical aspects of the medication reconciliation process resulting in better compliance and more accurate reconciliation.

### Limitations

There are some limitations to this review. Although we aimed to maximize coverage of the grey literature through the inclusion of all publication types, including conference proceedings, we only included studies written in English or French. As such, there is potential for publication bias. In addition, given the heterogeneity of the studies ultimately included in our review, we were unable to effectively combine the results across studies, and thus the results across studies have been described qualitatively. We also did not quantitatively assess the studies for risk of methodological bias, again, due to the heterogeneity of studies in terms of study populations, outcomes measured and educational interventions.

## Conclusion

Preventable adverse drug events cause a significant burden on the health care systems worldwide [3]. The process of medication reconciliation is mandated in North America in an effort to decrease such medication errors and improve quality of care and patient safety. However, compliance and accurate reconciliation remains a challenge in most health care institutions [3,4,23]. In addition, although medical trainees are often the frontline health providers responsible for medication reconciliation on admission, transfer and discharge of the most vulnerable patients, they currently have little formal training on the reconciliation process. Instead, they derive much of their learning, when possible, through an informal curriculum driven by hospital-based quality-improvement initiatives.

The findings of this review indicate that a combination of didactic sessions, role-play exercises and experiential learning at the undergraduate level may be effective in educating medical students in the how to conduct medication reconciliation [26-29]. None of the studies concerning the education of medical students relied solely on didactic methods. In the education of medical residents or postgraduates, the studies included in this review also suggest that experiential learning methods should be an important part of training students in how to carry out medication reconciliation. The improvements made by the residents participating in these studies, however, suggest that there is room for improvement. In the study conducted by Mann et al., while 61% reported an increase in confidence after the educational exercise involving a 15-minute standardized patient interview, only 37.2% of the residents were considered ‘high performers’ in carrying out effective medication reconciliation [30]. The authors also noted a significant advantage in the level of skills of primary care trainees compared to residents of other specialties [30]. In the studies conducted by both Young et al. (2011, 2012), participating residents also indicated that their skills in conducting medication reconciliation had not significantly improved [31].

In an attempt to improve the education of medical residents in medication reconciliation, educators should consider incorporating other teaching methods into postgraduate curricula. Integrating resident representation during implementation of medication reconciliation practices and follow-up on their feedback also demonstrates promising results in compliance and accuracy rates, although several challenges lie ahead in effectively integrating residents in these quality improvement initiatives. This includes a lack of understanding about preventing adverse drug events, tight resident schedules, lack of structured reconciliation training programs, rotation at several health care institutions, and limited financial resources.

The most appropriate approach to tackling such barriers includes the early education of trainees even at the pre-clinical level on patient safety issues with a strong in-context clinical curriculum in the more senior training years. Furthermore, house staff involvement in conception, design, implementation and improvement of the medication reconciliation process is a vital step to ensure compliance and usability. The involvement of trainees in the development of quality improvement programs is important to ensure not only compliance, but also accurate feedback of such programs. Training programs should also create a non-judgmental, safe environment, which would contribute to the trainees’ willingness to disclose medical errors. Finally, the use of simulation based medical education should be an integral part of the curriculum by boosting trainees’ performance without compromising patient care.

## Appendix

### MEDLINE Search strategy

1. Medical Records Systems, Computerized/
2. Medication Errors/
3. Medication Reconciliation/
4. “Continuity of Patient Care”/
5. Medication Systems/
6. Drug Prescriptions/
7. Medication reconciliation.tw
8. or/1-7
9. Patient Discharge/
10. Patient discharge.tw
11. or/9-10
12. 12.Students, Medical/
13. “Internship and Residency”/
14. medical trainee.tw
15. Education, Medical, Graduate
16. Education, Medical, Continuing
17. Education, Medical, Undergraduate
18. or/12-17
19. 19.8 and 11 and 18
